# Public Works Programmes and Cooperation for the Common Good: Evidence from Malawi

**DOI:** 10.1057/s41287-022-00525-1

**Published:** 2022-04-23

**Authors:** Stefan Beierl, Marina Dodlova

**Affiliations:** 1 Chair of Development Economics, University of Passau, Innstrasse 29, 94032 Passau, Germany; 2 CESifo, Munich, Germany

**Keywords:** Public works, Cash-for-work, Social protection, Cooperation for the common good, Coordination, Community meeting, Voluntary contributions

## Abstract

This paper investigates the relationship between Malawi’s largest and oldest public works programme (PWP) and social cohesion, specifically within-community cooperation for the common good. Using both primary and secondary data, we show that public works are associated with higher coordination activities and higher voluntary (unpaid) contributions to public goods, along both vertical ties (between community members and local leaders) and horizontal ties (among community members). Especially for school-building activities, voluntary inputs in the form of labour and other in-kind contributions are higher in the presence of the PWP. Our results contribute to a better understanding of the link between social protection programmes with community-driven features and social cohesion.

## Résumé

Cet article étudie la relation entre le plus grand et le plus ancien programme de travaux publics du Malawi, d’une part, et la cohésion sociale, d’autre part, et plus particulièrement la coopération au sein des communautés pour le bien commun. Grâce à des données à la fois primaires et secondaires, nous montrons que les travaux publics sont associés à des activités de coordination plus nombreuses et à une aide bénévole (non rémunérée) plus importante au niveau des biens publics, à la fois pour les liens verticaux (entre les membres de la communauté et les dirigeants à l’échelle locale) et les liens horizontaux (entre les membres de la communauté). L’aide bénévole sous forme de main-d'œuvre et d'autres contributions en nature est plus importante en présence du programme de travaux publics, en particulier pour les activités de construction d'écoles. Nos résultats contribuent à une meilleure compréhension du lien entre les programmes de protection sociale à caractère communautaire et la cohésion sociale.

## Introduction

It has long been understood that social cohesion is needed for societies to be successful (Knack and Keefer 1997; Nosratabadi et al. 2020).^1^ Strong reciprocal relationships and joint community activities can serve as a cushion that insures people against shocks by providing mutual financial and social support during times of need. They are also important in the face of large collective challenges such as the COVID-19 pandemic, climate change and scarcity of common resources. One potential channel to build or maintain social cohesion is social protection (Burchi et al. 2022). In developed countries, scholars have predominantly found a positive relationship between social protection (in terms of welfare state generosity) and social cohesion (Ferragina 2017; Kumlin and Rothstein 2005; Rothstein 2001). Yet, evidence on the link between social protection and social cohesion in developing countries is limited and mixed (Burchi et al. 2020; Burchi and Roscioli 2022; Strupat, 2021). Moreover, social protection programmes have been typically discussed in the context of reducing poverty and improving human capital, and well-being. However, their effects on social cohesion are understudied. We contribute to the literature by investigating the relationship between Malawi’s largest and oldest social protection programme, the Malawi Social Action Fund (MASAF) Public Works Programme (PWP), and social cohesion. More specifically, we assess an effect of public works on social cohesion using two independent primary and secondary data sources, several empirical strategies and various measures of within-community cooperation.

We rely on the theoretical framework suggested by Leininger et al. (2021) that distinguishes three attributes of social cohesion: cooperation for the common good, inclusive identity and trust. In this paper, we focus on the first attribute. Our notion of cooperation comprises within-community coordination and contributions to local public goods. We measure *coordination* through meetings linked to addressing common needs and *contributions* through voluntary unpaid labour contributions to communal activities and other in-kind contributions for community purposes. Following Leininger et al. (2021) we stress three important features of cooperation for the common good. First, one actor’s actions for the community’s benefit should be costly but voluntary. Second, “cooperation takes place despite incentives for non-cooperation”. Third, we focus on “actual cooperation” rather than declared willingness to cooperate. In line with Leininger et al. (2021), we further distinguish *vertical* cooperation (between citizens and traditional/local leaders) and *horizontal* cooperation (among citizens).

The majority of existing studies on the relationship between social protection and social cohesion concern cash transfer programmes, whereas PWPs, another popular social protection instrument, have so far received less attention. PWPs are transfer programmes that require participants to work on public projects for wages that are deliberately set below the market rate or at the level of the minimum wage. This principle is meant to ensure that only those in need enrol while the non-poor are discouraged from programme participation. Therefore, PWPs have many important features in common with both transfer-based social protection interventions like cash transfer programmes and community-driven development (CDD) projects that promote the provision of public goods. The crucial difference to the former is the work component and to the latter the remuneration of work. Evidence regarding their respective effect on social cohesion is mixed for both cash transfers and CDD projects.

Most studies of cash transfers demonstrate a positive effect on different outcomes of social cohesion (Attanasio et al. 2015; Barca et al. 2015; Camacho 2014; Evans et al. 2019; Pavanello et al. 2016; Valli et al. 2019), although a few studies find no effects (Veras Soares et al. 2010) or even unintended negative effects due to perceived unfairness, especially related to targeting (Adato 2000; Adato and Roopnaraine 2004; Cameron and Shah 2014; Devereux et al. 2017; Kardan et al. 2010).

Specifically on group membership, studies of cash transfer programmes in Peru (Camacho 2014) and Paraguay (Veras Soares et al. 2010) do not detect any effects.^2^ However, cash transfer beneficiaries in Columbia are more cooperative in public good games (Attanasio et al. 2015). In Tanzania, beneficiaries report a higher willingness to contribute but actual participation in community work does not increase (Evans et al. 2019). Qualitative evidence from Mexico’s conditional cash transfer programme PROGRESA suggests that some non-beneficiaries reduce their contributions because they consider them the task of the beneficiaries (Adato 2000). In short, there are no strong indications that programmes without a work component commonly result in large changes in coordination for the common good.

However, the work activities of PWPs require close contacts among community members and might stimulate further cooperation for the common good outside the framework of the programmes. Indeed, for a CDD project in Morocco, Nguyen and Rieger (2017) find increased contributions in public goods games with modest stakes (around 120% of average daily wage). Breuer and Asiedu (2017) show for urban settings in Togo that gender-targeted employment interventions can foster community participation, especially among women. However, two “synthetic” reviews, including one with a focus on Africa, conclude that very few of the evaluated CDD projects have positive effects on cooperation for the common good including meeting attendance and participation (King et al. 2010; White et al. 2018). Most CDD projects also appear to primarily rely on existing social cohesion rather than building it, including in Malawi (Vajja and White 2008). Equally, Khwaja (2009) argues that any development project can be successfully implemented if failures in the design of the project are compensated by revealed social cohesion.

There are only a few studies which directly investigate the link between PWPs and social cohesion. Interestingly, they find positive effects on horizontal dimensions of social cohesion. Quasi-experimental evidence for India’s National Rural Employment Guarantee Act (NREGA) scheme, the largest PWP in the world, suggests that social networks have been intensified in programme villages (Bhuwania et al. 2016). In a refugee context in Jordan, cash-for-work programme beneficiaries that participate in waste-related public works report a higher willingness to voluntarily cooperate in the waste sector in the future (Loewe et al. 2020). However, to our best knowledge, there are no quantitative studies that investigate the relationship between public works and social cohesion in the African context; especially none that investigate the heterogeneity of cooperation for the common good by sectors.

Another contribution of our paper is the combination of results obtained for a similar set of outcomes using both primary and secondary data. The primary data was collected in two waves (2017 and 2019) from 500 randomly selected households in three clusters of villages where the MASAF-4 PWP was implemented. The secondary data is composed of the Integrated Household Surveys (IHS) conducted by the World Bank in 2010, 2013 and 2016. We first present results from the fixed effects panel data analysis for the primary data sample at the household level and for 102 enumeration areas (EAs) from the IHS at the community level. In addition, we disentangle in both samples the sectors in which cooperation takes place and which are outside of the PWP framework (e.g. school, health, and land management).

As in the existing studies, endogeneity concerns prevent a rigorous identification of causal PWP effects on social cohesion because neither the assignment of the MASAF PWP to communities nor the enrolment of households in the programme is randomised. While our estimates could still be biased due to unobserved factors that affect both PWP status and cooperation for the common good, all the results do include fixed effects for the unit of analysis (households or communities) and a variety of control variables. This ensures that the comparison is between similar communities or households, and that the estimates reflect the effect of PWP participation status rather than the effects of community or individual attributes. To address the endogeneity problem, we additionally perform a difference-in-difference (DID) with matching for the primary data sample and an instrumental variable (IV) approach for the secondary data sample. Given a geographical targeting and vast expansion of the programme after 2010, we argue that the presence of the PWP at the community level is more likely exogenous for a particular community as exposure to the programme is determined at the more aggregate level. Therefore, in the IHS sample, we instrument a number of respondents who report that they participate in the PWP programme in a particular community with a dummy denoting the presence of the PWP based on the community responses. These empirical strategies applied in both primary and secondary data analyses allow us to reveal a causal effect of public works on social cohesion.

We find a significant positive effect of PWPs on cooperation for the common good which is robust across different outcomes and samples. Specifically, we find that PWPs are positively associated with overall coordination among community members as well as top-down and bottom-up cooperation in total and in specific sectors like agriculture, public transportation and bridges, school-building activities and care. In the presence of PWPs, voluntary contributions are also higher in specific sectors, especially for school-building activities where we find a positive association in both samples. Most sectors for which we find increased cooperation are not the sectors in which PWP activities typically take place. Therefore, we can rule out in most cases that observed associations are merely a mechanical effect of cooperation linked to the PWP implementation.

Our results contribute to a better understanding of how social protection programmes with community-driven features are linked to social cohesion across sectors and along both horizontal and vertical lines in a developing country context, particularly in a setting with a relatively homogenous population unaffected by violent conflict.

The remainder of the paper is organised as follows. Section 2 describes the Malawian context. Section 3 presents the data, sample properties and methodology. Section 4 reports and discusses the results. Section 5 concludes.

## Malawian Context

Malawi is a peaceful and politically relatively stable country that made notable improvements in some dimensions of human development in recent years, but poverty and food insecurity remain persistently high. Around half of the population continues to live below the national poverty line (World Bank 2020). 20.1% lived in extreme poverty in 2016/2017, somewhat down from 24.5% in 2010/2011. As a means to bolster the food security of poor households with excess labour capacity, PWPs have been implemented in Malawi since 1996. It has since been the main social protection instrument accessible to the working age population. The PWP under the MASAF has been by far the longest-running and biggest PWP in the country. Funding for the MASAF PWP comes mainly from the World Bank, but it is implemented through government structures. Phase 3 (2002 to 2015) and Phase 4 (2016 to 2018) of the programme are the main interventions in our analysis.

The MASAF PWP operates nationwide. According to the nationally representative IHS data, in 2013 and 2016 70% of the survey clusters were exposed to the PWP. Funding is allocated to each district in proportion to population size and poverty levels. District officials are then expected to use the same criteria to target specific communities in their districts. Based on findings from other countries (Tavits 2009), politics can play a role in the geographical targeting of antipoverty programmes, but we have no information whether it does in Malawi. Due to the decentralised allocation procedure, it is unlikely that bias would be systematically based on the same factors across the country.

At the community level, the programme targets the poor and vulnerable with labour capacity. Participants are selected via community-based targeting without clear and standardised procedures and criteria. Officially, the programme foresees wealth rankings that are publicly discussed in community meetings. In practice, the extent of community involvement varies and local traditional leaders (chiefs) often play a key role, sometimes in conjunction with the Village Development Committee (VDC). Access must be rationed because demand usually exceeds the number of spots available in the programme (Beegle et al. 2017). Yet, there are no strict or clear eligibility criteria to guide the rationing, which gives a lot of discretion to local decision making. Studies of the MASAF PWP (Beegle et al. 2017) and other programmes in Malawi with a similar targeting approach (Basurto et al. 2020) find room for improvement in reaching the food insecure and evidence of some nepotism linked to the central role of local leaders in the process, but no signs of severe mistargeting.

Since 2012, the MASAF PWP offers participants up to 48 workdays per year and prior to that only up to 12 workdays (Beegle et al. 2017). The daily wage rate was occasionally adjusted upward to keep up with inflation and varies around the equivalent of somewhat less than 1€ per day. The biggest change from MASAF-3 to MASAF-4 was the shift from selecting participants anew for each work cycle to a 3-year targeting period. Hence, predictability of income from the PWP for those selected increased, but those not initially selected could not count on getting access to the PWP within that 3-year period. Moreover, the focus of work activities under MASAF-4 shifted somewhat towards environmental activities such as afforestation and soil and water conservation, but classical infrastructure projects such as road work continued to be undertaken as well.

Several studies investigated whether the MASAF PWP achieves its core objectives, in particular, food security (Beegle et al. 2017; Bloom et al. 2005; Chirwa et al. 2002).^3^ To our knowledge, the relationship between the MASAF PWP and social cohesion has not been studied for any phase.

## Data and Methodology

### Primary Data

The two-period panel data were collected from randomly sampled households in three purposely selected village clusters (*catchments* hereafter) where the implementation of the MASAF-4 PWP had started in early 2016. All catchments are located in Malawi’s Central Region, one in Mchinji District and the other two in neighbouring Kasungu District. The first wave was conducted in February 2017. In terms of types of public works activities, there were subprojects on afforestation, land management, and irrigation in all catchments, and additionally on roads in two of them.

The decision which households got to participate in the PWP was the outcome of the regular targeting process that took place in late 2015 and was, thus, not randomly assigned. In each catchment, the random sample of households was stratified by PW status, such that half of it comprised households participating in the PWP at the time and the other half of households not participating in the PWP at the time.

The second round took place 2 years later in the same month to ensure that seasonal variation does not bias the responses. Of the 616 respondents interviewed in the first round, 500 respondents could be re-interviewed. We balance the panel by omitting attrited households from the sample because for our empirical approach that relies on within-unit variation we need observation units that were observed in both waves.

The primary data allows us to investigate the relationship between participation in the MASAF-4 PWP and contributions for the common good, specifically voluntary unpaid labour contributions to community works. Note that *community works* in this paper denotes voluntary unpaid collective work outside the framework of the PWP, in contrast to *public works* which denotes remunerated collective work by PWP participants as part of the MASAF PWP. As shown in Table 1, we use the total number of workdays across all sectors and six sectoral dependent variables. The unit of these variables are ‘workdays in the past twelve months’. A workday in the questionnaire was not defined as a certain number of working hours. Instead, respondents were asked to report the number of days on which they contributed some work. We have no reason to believe that the average number of working hours per day substantially and systematically differed by PW status or location. In the preferred specifications, we winsorise all dependent variables of the primary data panel at fraction 0.98 to avoid distortions by outliers.^4^ Table 1 presents descriptive statistics of key household characteristics that serve as control variables in the empirical analysis.

**Table 1:** Descriptive statistics of the primary data

	Wave 1		Wave 2	
	Non-PW	PW	Non-PW	PW
Voluntary labour contributions to community works				
Number of workdays in last 12 months on […]				
All sectors combined	5.60	6.62	10.69	13.45
	(10.4)	(12.4)	(15.5)	(19.1)
Afforestation	0.05	0.90	0.68	2.03
	(0.5)	(4.5)	(4.5)	(7.3)
Land conservation	0.01	0.30	0.25	0.28
	(0.2)	(2.8)	(2.9)	(2.8)
Nursery/seedling production	0.00	0.12	0.31	1.62
	(0.0)	(1.8)	(3.2)	(6.8)
Road work	0.62	0.58	2.14	2.22
	(3.6)	(3.5)	(7.1)	(6.4)
Non-road construction	3.25	2.62	7.57	7.81
	(7.3)	(6.3)	(13.4)	(13.9)
School-related activities	2.25	2.49	7.11	7.46
	(6.4)	(7.0)	(13.4)	(13.7)
Control variables				
Household size	5.32	5.70	5.23	5.77
	(2.3)	(2.2)	(2.2)	(2.1)
Age of household head (in years)	42.20	43.07	43.50	45.29
	(15.8)	(14.7)	(15.2)	(14.6)
Married household head	0.80	0.88	0.77	0.87
Maximum education attained by head or spouse				
Primary completed	0.32	0.41	0.32	0.39
Secondary completed or more	0.05	0.10	0.06	0.09
Head or spouse with disability or chronic illness	0.11	0.08	0.28	0.26
Business or wage employment	0.29	0.31	0.26	0.23
Number of seven productive assets owned	0.92	1.12	1.14	1.42
	(1.0)	(1.1)	(1.2)	(1.2)
Number of 14 domestic assets owned	3.46	3.96	3.61	4.41
	(2.0)	(2.2)	(2.0)	(2.2)
Number of months with not enough food, last 12 months	3.63	3.83	2.90	2.30
	(2.7)	(3.0)	(2.6)	(2.4)
Observations	234	266	218	282

The reported values are the means with the standard deviation (SD) in parentheses below for non-binary variables. PWP participants are not necessarily the same across waves because some respondents dropped out of the programme and others newly entered between Waves 1 and 2. The sample size of each column group is reported in the last row. The dependent variables are winsorised at fraction 0.98. This corresponds to how the dependent variables are used in the preferred empirical specifications

### Secondary Data: IHS Panel

For the secondary data analysis, we construct a panel from the World Bank’s IHS, which tracks the life conditions of Malawian households. Social cohesion indicators in the IHS data are only available at the EA level, so our panel contains 102 EAs interviewed in three waves (2010, 2013 and 2016).

From the community questionnaire, we extract information on needs that community members have expressed in the last 3 years, whether any meeting activities to address them took place (villagers approaching local leaders, local leaders organising community meetings, or community members meeting without local leaders), and whether this was successful, whether any voluntary contributions were made by community members including time serving in committees, material and other in-kind inputs, whether community members belong to any groups and how frequent were meetings of these groups. We aggregate needs expressed in the following ten sectors: agriculture/livestock, maize mills, schools, health, care,^5^ public transportation, roads, bridges, piped water/boreholes, law enforcement and others. All needs indicate both construction and maintenance/improvement.

Our measures of community social cohesion are close to Leininger et al. (2021) as we also look at the initiative to organise meetings, group membership, and voluntary contributions by community members to the common good.

According to Gugerty and Kremer (2008) increased community interactions are not necessarily a sign of increased social cohesion because the need for them may arise from conflicts and dissatisfaction. Yet, in our setting, the original survey questions are phrased such that they mainly seem to capture constructive efforts to address communal needs. In addition, we interpret somewhat ambiguous outcomes (*approaching village head*, *organising community members*, *meetings among community members*) jointly with less ambivalent measures of coordination (*successful actions*). According to the survey guidelines, the community questionnaire was answered by ‘a group of several knowledgeable residents’ of the EA, often including the village headmen and other local leaders. Although there is some risk of misreporting if the residents wanted to show themselves in a better light, we cannot think of plausible reasons why such misreporting would be more or less probable or specific in communities with the PWP presence. Hence, it is unlikely to bias our results.

Information on MASAF PWP coverage is available in the community questionnaire and in the household questionnaire. We first construct a dummy denoting whether the MASAF-4 PWP employs people in the community based on the community questionnaire. Second, we aggregate information from the household questionnaire about households’ public works status into two indicators of PWP coverage at the EA level: the total number of PWP participants in an EA and the share of respondents in an EA that report to participate in the PWP.^6^

The set of control variables contains basic community characteristics like rural/urban location, population, number of households, major religions, common marriage types, number of polygamous households, and whether descent is traced through the mother or father.

The descriptive statistics of key variables are summarised in Table 2. Initiated meetings, vertically and horizontally, as well as successful actions take place, on average, in around three out of four EA-years and approximately two sectors. Voluntary contributions are most frequent in the form of spending time serving in committees, closely followed by providing material inputs and less often other in-kind contributions. Regarding the sectoral composition, vertical or horizontal meeting activities most frequently aim to address needs related to school (50% of the EA-years), closely followed by roads and water. Meeting activities concerning transport/bridges and health were also relatively common (28% and 21%, respectively).

**Table 2:** Descriptive statistics of the IHS data

	Mean	SD	Min	Max
Coordination				
Approaching a village head (bottom-up)	2.67	2.63	0	20
Organising community members (top-down)	2.66	2.65	0	20
Meetings among community members (horizontal)	2.34	2.46	0	21
Successful actions	2.01	2.17	0	17
Number of groups	28.59	44.59	0	547
Number of members in all groups	857.87	2959.80	0	41,910
Number of female members in all groups	510.74	1606.51	0	20,324
Number of members under 30 in all groups	406.35	1543.90	0	19,336
Maximal frequency of meetings	58.67	53.26	4	365
Meeting intervals				
Daily	0.03	0.16		
Weekly	0.92	0.28		
Monthly	0.92	0.27		
Quarterly	0.40	0.49		
Semi-annual	0.10	0.30		
Annual	0.06	0.23		
Contributions				
Time serving in committee	1.18	1.31	0	6
Material inputs	0.90	1.18	0	6
Other in-kind inputs	0.54	0.94	0	6
PWP coverage				
MASAF PWP operates in community (EA-level response)	0.56	0.50		
Household-level responses (aggregated to EA level)				
Number of PWP participants	1.65	2.18	0	10
Share of respondents that participate in PWP	0.10	0.12	0	0.63
Control variables				
Rural location	0.72	0.45		
Total population	7444.14	16,490.11	92	200,000
Total number of households	1455.64	2747.93	10	35,000
Descent traced through father	0.16	0.37		
Descent traced through mother	0.64	0.48		
Number of polygamous households	98.91	611.20	0	9000

People are active in communal activities, with an average of 30 groups and about 857 members in total per community. About half of them are young people under 30 and a bit more than a half are women. Maximum frequency of group meetings is slightly above one meeting per week. Holding meetings weekly or monthly is most common.

The MASAF PWP operated in 56% of the EA-years, according to community-level information. The household-level information suggests the presence of the PWP for a similar share of EA-years, measured by whether at least one respondent in an EA-year reported to be a PWP participant. However, this household-level measure matches the community level information for only about 70% of the EA-years. We argue that misreporting might be a reason of such discrepancy, and misreporting by the group that responded to the community questionnaire seems less likely than misreporting at the household level. In addition, in view of that only a few households were randomly selected in each EA to be interviewed in the IHS, we argue that PWP coverage based on households’ responses is probably highly underestimated. However, using the latter as an endogenous variable in an IV approach helps to address such measurement error.

## Methodology

We pursue different empirical strategies. As a starting point, we use a canonical panel data model with fixed effects. Depending on the sample, we estimate that model at the EA level (IHS panel) or at the household level (primary data sample). The basic econometric specification at the EA level for the IHS panel is as follows:

1 $${Y}_{ijt}=\mathrm{\alpha }+ \beta {\mathrm{PWP}}_{ijt}+ \sum {\gamma }_{k}{X}_{ijt}^{k}+{v}_{i}+{\varphi }_{j}+{\delta }_{t}+{\varepsilon }_{it},$$

where ${Y}_{it}$ denotes the respective social cohesion indicator for EA *i* at period *t*. ${\mathrm{PWP}}_{it}$ is a dummy whether the MASAF PWP is present in EA *i* in district *j* at period *t*. *X*^*k*^_*ijt*_ is the vector of all other control variables listed in Sect. 3.2.^7^ EA fixed effects refer to *ν*_*i*_ and capture particular time-constant EA characteristics. We also include district fixed effects to take into account time-constant district characteristics.^8^ Year effects denoted by *δ*_*t*_ capture common shocks and time trends for all EAs. The error term that captures all omitted variables and random errors is *ε*_*ijt*_*.* The standard errors are heteroscedasticity-robust and clustered at the EA level.

In the case of the primary data panel, ${Y}_{ijt}$ denotes the respective social cohesion indicator for household *i* in district *j* at period *t*. ${\mathrm{PWP}}_{ijt}$ is a dummy whether a member of the household is a PWP participant at period *t*. *X*^*k*^_*ijt*_ is the vector of all other control variables listed in Sect. 3.1. Household fixed effects refer to *ν*_*i*_ and year effects are denoted by *δ*_*t*_. District fixed effects are omitted in this specification. *ε*_*it*_ is the error term. The reported standard errors are heteroscedasticity-robust but not clustered or bootstrapped because it is not clear whether any alternative would be more accurate.^9^ With just three catchments and a moderately high, and widely varying, number of observations per catchment, clustering standard errors at the catchment level is not recommended (Cameron and Miller 2014; Canay et al. 2017, 2019; MacKinnon and Webb 2018; Roodman et al. 2019).^10^

The fixed effects panel analysis allows capturing within-unit variation and mitigating endogeneity concerns linked to non-random allocation of the PWP to communities (IHS panel) and non-random selection of PWP participants within communities (primary data panel). Our within-unit estimates could, however, be biased by unobserved factors that affect both PWP status and cooperation for the common good.

To address this concern, we perform two other strategies. First, we apply a DID approach with kernel matching for a reduced sample of the primary data. We drop *Always-takers* and *Dropouts* and leave in the sample only *Entrants* and *Never-PW* households. *Entrants* are households that did not participate in a PWP during Wave 1 but participated only during Wave 2. *Never-PW* households were not PWP beneficiaries during either of the two waves. The group of *Entrants* is of particular interest to us because they were observed before and after joining the PWP so that Wave 1 can be regarded as the baseline and the *Never-PW* households as the control group. In this specification, we keep household and wave fixed effects as well as all control variables. The main covariates of interest are then the *Entrants* dummy and the interaction term between the *Entrants* dummy and the second wave dummy. We believe that the problem of self-selection into PWP participation is mitigated as we include household fixed effects and additional controls to capture household characteristics. Additionally, we apply a DID only with the kernel matching procedure to ensure that *Entrants* and *Never-PW* households have similar characteristics. We also perform a balance test for two groups in 2017.

To address the endogeneity concern in the secondary data sample, we employ the IV approach. As described in Sect. 2, the MASAF PWP is meant to be allocated to communities following pro-poor geographical targeting, but to the best of our knowledge, the specific criteria used for this process at the district or sub-district level are unclear. And while we cannot say anything about why a specific EA was included into the MASAF PWP or not, we can clearly attribute the increase from 22 to 70% in the share of EAs covered between 2010 and 2013 to the nationwide scale-up of the MASAF PWP in the wake of the large currency devaluation in 2012 (Beegle et al. 2017). This can be regarded as an exogenous shock from the perspective of the specific EAs that benefited from this scale-up. We can use this fact and employ EAs exposure to the PWP status as an instrument for an individual PWP status of the IHS respondents. As the unit of analysis is an EA, our endogenous variable is a number or a share of respondents in an EA who report that they participate in the MASAF PWP.^11^

The final argument to the plausibility of our results relies on cross-validating findings across the empirical strategies and across the samples. In addition, the sample-specific endogeneity concerns are so different in primary and secondary data that it becomes unlikely that the bias would systematically be in the same direction across the samples and the outcomes.

## Results

In this section, we report the results on the relationship between PWPs and social cohesion. We anticipate that PWPs can enhance vertical and horizontal coordination as well as voluntary contributions to local public goods (outside the PWP framework). In the following, the results from both primary and secondary data analyses are presented for these two components.^12^

### Coordination

Table 3 reports results for coordination from the IHS panel analysis. We report disaggregated results by sector and by dimension of interactions (vertical versus horizontal). Fixed effects estimations are presented in odd columns and IV estimations are presented in even columns. The treatment variable in the FE specifications is a dummy for the presence of the MASAF PWP in an EA based on the community responses. The endogenous variable in the IV specifications is the number of households in an EA who report that they participate in the MASAF PWP (based on the individual responses).^13^ The instrument is a dummy for the presence of the MASAF PWP in an EA based on the community responses. For all sectors, the dependent variable is a number of sectors (maximum 10) in which YES is reported for the respective social cohesion indicator. For each specific sector, the dependent variable is a dummy for YES for the respective social cohesion indicator. Columns 1–2 and 3–4 present top-down and bottom-up interactions between community members and village headman or other local leaders. Columns 5–6 report the results for horizontal coordination measured by the initiation of meetings among community members. Columns 7–8 show whether the meetings were deemed successful in taking the necessary steps to address the needs in the respective sector(s).

We find that all four indicators of social cohesion are higher in the presence of the PWP. These results are confirmed in both FE and IV estimations except horizontal interactions where only FE estimate is significant at the 10% level, and the IV estimate remains significant only at the 15% level. However, the effect sizes between FE and IV are comparable for all four outcomes. Bottom-up interactions take place on average in 0.7 more sectors and top-down interactions take place on average in 0.9 more sectors than in the absence of the PWP. Horizontal interactions are less significant but are increased in 0.5 more sectors than in the absence of the PWP. All meeting activities are successful in 0.8 more sectors.

We find some significant positive effects of public works on coordination in four sectors: agriculture, schools, public transportation/bridges and care.^14^ However, the IV results are strongly significant only for two sectors, agriculture and transport/bridges. Schools and care are characterised by an increase in coordination significant only at the 15% level. Regarding agriculture, vertical interactions are higher by about 13% in the MASAF PWP communities. Horizontal meetings take place more often by about 11% in such communities. Regarding schools, bottom-up and top-down interactions are higher by about 14–18%. Regarding transport/bridges, we find a positive effect of exposure to the MASAF PWP for both vertical interactions and horizontal meetings, with effect sizes between 10 and 23%. Regarding care, we find a statistically significant effect only for top-down interactions (10%) (Table 3).

**Table 3:** Vertical and horizontal coordination: IHS panel

	Approaching a village head (bottom-up)		Organising community members (top-down)		Meetings among members (horizontal)		Successful actions	
	FE	IV	FE	IV	FE	IV	FE	IV
	(1)	(2)	(3)	(4)	(5)	(6)	(7)	(8)
All sectors	0.619*	0.754*	0.827**	1.006*	0.528*	0.642	0.723**	0.880*
	(0.314)	(0.490)	(0.325)	(0.562)	(0.312)	(0.455)	(0.295)	(0.521)
Agriculture	0.100**	0.132*	0.132***	0.177**	0.094**	0.121*	0.091**	0.117*
	(0.045)	(0.075)	(0.045)	(0.089)	(0.042)	(0.068)	(0.043)	(0.066)
Schools	0.135*	0.184	0.141*	0.185	0.099	0.153	0.156**	0.233
	(0.076)	(0.136)	(0.077)	(0.136)	(0.078)	(0.126)	(0.076)	(0.146)
Transport	0.104*	0.187	0.139*	0.226*	0.152**	0.239*	0.124*	0.221*
and bridges	(0.075)	(0.119)	(0.075)	(0.133)	(0.075)	(0.133)	(0.073)	(0.127)
Care	0.066	0.090	0.099*	0.136	0.072	0.097	0.080*	0.108
	(0.063)	(0.094)	(0.061)	(0.102)	(0.056)	(0.088)	(0.052)	(0.085)
Observations	278	278	278	278	278	278	278	278
EA FE	YES	YES	YES	YES	YES	YES	YES	YES
Wave FE	YES	YES	YES	YES	YES	YES	YES	YES
Controls	YES	YES	YES	YES	YES	YES	YES	YES

Fixed effects estimations are presented in odd columns. The treatment variable is a dummy for the presence of the MASAF PWP in an EA based on the community responses. IV estimations are presented in even columns. The endogenous variable is a number of households in an EA who report that they participate in the MASAF PWP (based on the individual responses). The instrument is a dummy for the presence of the MASAF PWP in an EA based on the community responses. For all sectors, the dependent variable is the number of sectors in which YES is reported for the respective social cohesion indicator. For each specific sector, the dependent variable is a dummy for YES for the respective social cohesion indicator. The control variables are rural location, total population and a number of households in an EA, descent tracing through mother or father, and number of polygamous households. All specifications include EA, district and time/wave fixed effects. Robust standard errors are in parentheses

***p < 0.01, **p < 0.05, *p < 0.1

For all three sectors, the higher meeting activities coincide with positive significant effects for meeting success. Recall that the assessment of meeting success was made by local key informants who might be the same people that were approached by their community members (bottom-up) or who organised their community members (top-down). Despite this caveat, we take the results for meeting success as consistent with interpreting intensified meeting activities as proxies for higher social cohesion rather than indications of unresolved conflicts or dissatisfaction. In agriculture and care, meetings are recognised as successful on average in 10% more cases in communities with the presence of the PWP. In transport/bridges and schools, the effects of the PWP presence on the successfulness of meeting activities are even higher and come up to 22%.

Additional significant results in the IHS panel refer to the higher number of groups or associations in communities with the presence of the MASAF PWP. This is confirmed by both FE and IV estimations. However, the total number of group members and the frequency of meetings are not significantly different in communities with and without the PWP presence.

Our findings contribute to the literature by showing that there are more coordination activities in the form of initiated meeting activities in the presence of the PWP. More specifically, the presence of the PWP is associated with more intensive bottom-up and top-down as well as horizontal interactions on initiating and organising meetings within communities. In addition, we find that the positive association is especially strong and robust in three specific sectors: agriculture, schools and transport/bridges.

### Contributions

The results for labour contributions and other in-kind contributions to public goods are summarised in Table 4. The first six columns present the results of the EA-level analysis based on the IHS panel where the outcomes are *time* spent on committees, *materials* and *other in-kind* contributions. Fixed effects estimations are presented in odd columns and IV estimations are presented in even columns. In the FE specifications, the treatment variable is a dummy for the presence of the MASAF PWP in an EA based on the community responses. In the IV specification, the endogenous variable is a number of households in an EA who report that they participate in the MASAF PWP (based on the individual responses). The instrument is a dummy for the presence of the MASAF PWP in an EA based on the community responses. For *all sectors*, the dependent variable is a number of sectors (maximum 10) in which the respective contribution was made. For each specific sector, the dependent variable is a dummy for YES for the respective type of contribution.

Columns 7–8 report the results of the household-level analysis based on the primary data panel. The outcome variable is a number of voluntary unpaid community workdays during the previous 12 months in any sector or in the respective sector. The treatment variable is a dummy denoting whether the household is enrolled in the MASAF-4 PWP. Column 7 shows the results of the FE specification. Column 8 contains the results of the DID specification for a reduced sample where only *Entrants* are compared with *Never-PW* households. For all sectors, there is a robust positive association between the MASAF PWP and all types of contributions. The results from the IHS panel show that in the presence of the PWP, time on committees is spent in about 0.7 additional sectors, materials are contributed in about 0.8 additional sectors and other in-kind contributions are made in 0.35 additional sectors. The results from the primary data panel suggest that when a household is enrolled in the PWP its members contribute almost 8 additional voluntary unpaid workdays (Table 4).

**Table 4:** Contributions

	IHS panel						Primary data panel	
	Time serving in committee		Material inputs		Other in-kind		Labour	
	FE	IV	FE	IV	FE	IV	FE	DID
	(1)	(2)	(3)	(4)	(5)	(6)	(7)	(8)
All sectors	0.611***	0.744*	0.712***	0.866*	0.323**	0.393	7.815**	7.861**
	(0.207)	(0.403)	(0.186)	(0.450)	(0.142)	(0.248)	(3.197)	(3.891)
Schools	0.119*	0.120	0.179**	0.241*	0.049	0.136	4.549*	6.550**
	(0.075)	(0.118)	(0.074)	(0.149)	(0.058)	(0.100)	(2.686)	(2.839)
Transport and bridges	0.117**	0.184*	0.055	0.145*	0.061*	0.028		
	(0.046)	(0.107)	(0.042)	(0.092)	(0.039)	(0.046)		
Care	0.091**	0.124	0.072*	0.098	0.061*	0.082		
	(0.043)	(0.079)	(0.041)	(0.072)	(0.037)	(0.060)		
Afforestation							3.418**	3.346**
							(1.583)	(1.596)
Non-road							4.465*	5.092*
construction							(2.562)	(3.112)
Observations	278	278	278	278	278	278	1000	468
Unit of analysis	EA	EA	EA	EA	EA	EA	Household	Household
Unit FE	YES	YES	YES	YES	YES	YES	YES	YES
Wave FE	YES	YES	YES	YES	YES	YES	YES	YES
Controls	YES	YES	YES	YES	YES	YES	YES	YES

Columns 1 to 6 report the results of the IHS panel analysis. Fixed effects estimations are presented in odd columns. The treatment variable is a dummy for the presence of the MASAF PWP in an EA based on the community responses. IV estimations are presented in even columns. The endogenous variable is a number of households in an EA who report that they participate in the MASAF PWP (based on the individual responses). The instrument is a dummy for the presence of the MASAF PWP in an EA based on the community responses. For *all sectors*, the dependent variable is a number of sectors in which YES is reported for the respective type of contribution. For each specific sector, the dependent variable is a dummy denoting YES for the respective type of contribution. The control variables are rural location, total population and a number of households in an EA, descent tracing through mother or father, and number of polygamous households. All specifications include EA and time/wave fixed effects. Columns 7–8 report the results of the primary data panel analysis. The treatment variable is a dummy denoting whether the household is enrolled in the MASAF-4 PWP. Column 7 shows the results of the FE specification. Column 8 contains the results of the DID specification where only *Entrants* are compared with *Never-PW* households. For *all sectors*, the dependent variable is a number of voluntary unpaid community workdays during the previous 12 months. To avoid distortion by outliers, these dependent variables are winsorised at fraction 0.98. The control variables are household size, education levels, age, head or spouse disabled, household head married, sum of productive assets owned, sum of domestic assets owned, employment and business status, and food gap. All specifications include household and wave fixed effects. Robust standard errors are in parentheses

***p < 0.01, **p < 0.05, *p < 0.1

Regarding specific sectors, we find a positive effect of the MASAF PWP presence on contributions in schools, transport/bridges, care, afforestation and non-road construction. For the two sectors where we have data from both samples, the results are consistent. There is significant positive association for the school sector and no significant association for the road sector. Across samples, outcomes, sectors and empirical strategies we do not detect any statistically significant negative association.

The sectoral results of the primary data panel analysis suggest that when a household is enrolled in the PWP its members contribute an additional 4.5 voluntary unpaid community workdays to school-related building activities, 3.4 workdays to non-road construction (which also comprises school-related construction), and 4.5 workdays to afforestation activities. These effects become a bit higher in the school and afforestation sectors if we compare only *Entrants* and *Never-PW* in DID settings. In school-building activities, individuals from households participating in the MASAF PWP contribute with 6.5 more workdays, in afforestation with 5 more workdays. No significant effects are observed for land conservation and seedling production/nursery.^15^

In the IHS sample, voluntary contributions are higher in communities with the presence of the MASAF PWP in schools, transport/bridges and care. The remaining sectors demonstrate less robust results across all three types of contributions. Regarding material inputs, the size of the effect ranges from 7% for care to 24% for schools. The school sector demonstrates the most robust results, which are strongly significant in both FE and IV specifications. Regarding time spent on committees, we find a significant positive increase of about 10% for all mentioned sectors in the FE estimations. In the IV estimations, the results are robust only for transport/bridges and somewhat less significant in care. The effects come to 18% and 13%, respectively. Regarding other in-kind contributions, only the transport/bridges and care sectors demonstrate significantly positive results. However, these results are not confirmed by the IV estimations.

The key insight of our findings is that PWPs can be associated with increased contributions to public goods, similar to what has been observed for some CDD projects (e.g. Nguyen and Rieger 2017). Specifically, we find this positive association both for the presence of a PWP (in the IHS panel analysis) and for household participation in the PWP (in the primary data panel analysis), for several sectors, especially the school sector, and for different forms of contributions (time, materials and labour).

## Conclusion

Public work programmes are promising for enhancing social cohesion because due to the work component they require a higher quantity and quality of interactions than other types of social protection. Therefore, it is relevant to know more about the relationship between PWPs and social cohesion. This paper contributes to this literature with evidence on the link between Malawi’s MASAF PWP and one attribute of social cohesion, namely cooperation for the common good within communities. The quantitative literature has so far been silent on these issues and there are, to the best of our knowledge, no studies on the African context. In awareness of the methodological challenges that complicate the investigation of social cohesion with the use of quantitative methods such as formulation of proper indicators, under and over reporting of actual behaviour, impossibility to reveal the respondents’ motivations, we cross validate the results using two independent data sources, different empirical strategies and various measures of within-community cooperation.

Our empirical analysis shows the positive and significant association between the presence of public work programmes and cooperation for the common good within communities. We demonstrate that the association is robust across specific sectors (especially schools) and along both horizontal and vertical lines. In addition, our results point to a potential causal effect of public works on this attribute of social cohesion.

Because social cohesion indicators in the secondary data are reported at the community level, we do not know whether our results are driven by PWP participants or even non-participants. However, in the primary data analysis we know precisely that increased voluntary contributions are provided by the MASAF PWP participants and these contributions are not related to public works. In the secondary data analysis, we cannot completely rule out that some elements of cooperation have directly to do with the public works activities because the IHS questions on social cohesion do not specify whether meeting activities and contributions are directly linked to the MASAF PWP or separate from it and there is no information in the IHS data about the sectors in which PWP activities took place in an EA. Yet, of the sectors for which we find positive significant effects, PWP activities take place typically only in one sector, namely transportation/bridges. For the other sectors, we can rule out that observed associations are merely a mechanical effect of cooperation linked to PWP implementation. Moreover, we know from the primary data, where we can disentangle voluntary unpaid labour contributions from public works activities, that the former are often not part and parcel of the latter (Beierl and Grimm 2018). So, we can expect the same to hold for the communities in the IHS sample.

The absence of a matching qualitative study to explore the potential implications of the quantitative findings is another limitation of this paper. Yet, the primary dataset contains some useful information from open-ended questions. All survey respondents were asked in a form of an open question which benefits the community derived from the PWP. Seven percent of respondents proactively reported improved community cohesion as one of the PWP’s benefits. The share of such responses among non-PW households was roughly equal to the share among PW households, which alleviates the worry that the responses mostly reflect cheap talk by PW households.^16^ Nevertheless, further qualitative research into the motives for cooperation would be needed to better understand how far the observed statistical associations can be interpreted as increased social cohesion driven by the PWP.

Another question to explore is whether the presence of PWPs is also associated with improvements in other attributes of social cohesion distinguished by Leininger et al. (2021). Specifically, participation in and satisfaction with the PWP might also improve within-community trust. The presence of the PWP programme could also lead to a more positive perception of the state. However, while the MASAF PWP is implemented through the state structures its funding comes mainly from the World Bank. Therefore, it is unclear who people assign praise to.

Investigating the channels through which social protection affects social cohesion is another direction for further research. The potential mechanisms that could affect the relationship are intensified contacts between PWP participants, labour remuneration, benefits from respective public goods, targeting perceptions and social pressure. Insights into these and other mechanisms may, for example, help to understand how to avoid unintended negative side effects of social protection programmes on social cohesion in developing countries. Accounting additionally for the perceptions and behaviour of non-participants in the context of social protection programmes would also help to comprehensively uncover the channels linking social protection and social cohesion.

Our paper provides insights that policy makers might achieve improved social cohesion as a positive side effect of policies that have other primary objectives. Public works, in particular, demonstrate potential in this respect. The strengthened social cohesion may serve as a basis for good community performance and successful collective actions, also in times of crises.

## Footnotes

1 Here and thereafter, we use the terms social cohesion and social capital interchangeably and draw on both strands of the literature.

2 In the secondary data sample, there are indicators that allow investigating the relationship between public works and group membership. We also do not find robust statistically significant effects for group membership. These additional results are available upon request.

3 Employing a randomised controlled trial, Beegle et al. (2017) do not find any evidence that the MASAF-3 PWP improved food security.

4 The results are robust to winsorising or truncating the dependent variables at different levels/fractions (available upon request).

5 Health denotes health centre/clinic/dispensary whereas care denotes community-based child care centre/nursery/orphanage.

6 In principle, there is also community-level information on the share of the population in the community that participates in the PWP (1/4, 1/2 or 3/4 and more, both for the female and male population). However, we do not use these indicators because their accuracy is questionable considering that data frequently show unrealistically high coverage rates of up to 3/4 of the population.

7 All specifications are also estimated without control variables. The results remain robust to non-inclusion of all control variables and are available upon request.

8 A few EAs cross district boundaries so EAs fixed effects do not fully cover the change in district characteristics.

9 However, we check that our results are robust to using different methods to calculate standard errors, particularly ordinary wild bootstrap.

10 Clustering at an intermediate level between the household and catchment is not an option either. If PWP subprojects had been allocated to specific villages, we could have clustered at the village level and, thereby, increased the number of clusters. However, there are not enough cases in our sample where the villages and subprojects overlap well enough to consider clustering at the village level as a viable alternative.

11 The standard tests show that the instrument is valid. The *F*-test of the first stage is 4.32.

12 We focus only on sectors with significant results. The remaining sectors’ results are available upon request.

13 The results with the share of households in an EA who report that they participate in the MASAF PWP (based on the individual responses) as an endogenous variable are consistent but of lower significance. They are available upon request.

14 By contrast, we do not find any robust effects for roads, health, water, maize, mills, and law enforcement. Such sectoral heterogeneity in the results might be explained by several reasons including, for example, involvement and specialisation of selected EAs in particular sectors.

15 The results based on the primary panel analysis are robust to truncating or winsorising the dependent variables at different levels/fractions, to restricting the sample to households where the same household member responded to the questionnaire in both waves, to not including control variables, and to using different methods to calculate standard errors.

16 Basic descriptive analysis of the characteristics of the respondents who mentioned this benefit does not suggest that the responses are driven by people from a particular locality or socio-demographic background.

## References

[CR1] Adato, M. 2000. *The impact of PROGRESA on community social relationships*. Report.

[CR2] Adato, M., and T. Roopnaraine. 2004. *A social analysis of the Red de Protección Social (RPS) in Nicaragua*. IFPRI Final Report (Issue December).

[CR3] Attanasio O, Polania-Reyes S, Pellerano L (2015). Building social capital: Conditional cash transfers and cooperation. Journal of Economic Behavior and Organization.

[CR4] Barca, V., S. Brook, J. Holland, M. Otulana, and P. Pozarny. 2015. *Qualitative research and analyses of the economic impacts of cash transfer programmes in Sub-Saharan Africa: Synthesis report*. FAO.

[CR5] Basurto MP, Dupas P, Robinson J (2020). Decentralization and efficiency of subsidy targeting: Evidence from chiefs in rural Malawi. Journal of Public Economics.

[CR6] Beegle K, Galasso E, Goldberg J (2017). Direct and indirect effects of Malawi’s public works program on food security. Journal of Development Economics.

[CR7] Beierl, S., and M. Grimm. 2018. *Mchinji pilot: Combining catchment management and public works—Qualitative Assessment Report*.

[CR8] Bhuwania, P., J. Heymann, A.N. Mukherji, and H. Swaminathan. 2016. *Public work programmes and social capital: An exploration of MGNREGA in India*.

[CR9] Bloom, G., W. Chilowa, E. Chirwa, H. Lucas, P. Mvula, A. Schou, and M. Tsoka. 2005. *Poverty reduction during democratic transition: The Malawi Social Action Fund 1996–2001.* No. 56; IDS Research Report. http://www.ids.ac.uk/files/Rr56.pdf. Accessed 30 June 2021.

[CR42] Burchi, F. and F. Roscioli. 2022. Can integrated social protection programmes affect social cohesion? Mixed-methods evidence from Malawi, *European Journal of Development Research*.

[CR41] Burchi, F., A. von Schiller, and C. Strupat. 2020. Social protection and revenue collection: How they can jointly contribute to strengthening social cohesion. *International Social Security Review* 73 (3): 13–32.

[CR40] Burchi, F., M. Loewe, D. Malerba, and J. Leininger. 2022. Disentangling the relationship between social protection and social cohesion. Introduction to the special issue. European *Journal of Development Research*. 10.1057/s41287-022-00532-2 PMC9059109 35527722

[CR11] Camacho, L.A. 2014. *The effects of conditional cash transfers on social engagement and trust in institutions: Evidence from Peru’s Juntos Programme*. DIE Discussion Paper 24/2014.

[CR12] Cameron C, Miller DL (2014). A practitioner’s guide to cluster-robust inference. Journal of Human Resources.

[CR13] Cameron L, Shah M (2014). Can mistargeting destroy social capital and stimulate crime? Evidence from a cash transfer program in Indonesia. Economic Development and Cultural Change.

[CR14] Canay IA, Romano JP, Shaikh AM (2017). Randomization tests under an approximate symmetry assumption. Econometrica.

[CR15] Canay IA, Santos A, Shaikh A (2019). The wild bootstrap with a “Small” number of “Large” clusters. SSRN Electronic Journal.

[CR16] Chirwa EW, Zgovu EK, Mvula PM (2002). Participation and impact of poverty-oriented public works projects in rural Malawi. Development Policy Review.

[CR17] Devereux S, Masset E, Sabates-Wheeler R, Samson M, Rivas A-M, te Lintelo D (2017). The targeting effectiveness of social transfers. Journal of Development Effectiveness.

[CR18] Evans DK, Holtemeyer B, Kosec K (2019). Cash transfers increase trust in local government. World Development.

[CR19] Ferragina E (2017). The welfare state and social capital in Europe: Reassessing a complex relationship. International Journal of Comparative Sociology.

[CR20] Gugerty MK, Kremer M (2008). Outside funding and the dynamics of participation in community associations. Political Science.

[CR21] Kardan, A., I. Macauslan, and N. Marimo. 2010. *Evaluation of Zimbabwe’s Emergency Cash Transfer (ZECT) Programme* (Issue July).

[CR22] Khwaja AI (2009). Can good projects succeed in bad communities?. Journal of Public Economics.

[CR23] King E, Samii C, Snilstveit B (2010). Interventions to promote social cohesion in Sub-Saharan Africa. Journal of Development Effectiveness.

[CR24] Knack S, Keefer P (1997). Does social capital have an economic payoff? A cross-country investigation. Quarterly Journal of Economics.

[CR25] Kumlin S, Rothstein B (2005). Making and breaking social capital: The impact of welfare-state institutions. Comparative Political Studies.

[CR26] Leininger, J., Burchi, F., Fiedler, C., Mross, K., Nowack, D., von Schiller, A., Sommer, C., Strupat, C., and Ziaja, S. 2021. *Social cohesion: A new definition and a proposal for its measurement in Africa*. DIE Discussion Paper 31/2021.

[CR27] Loewe, M., T. Zintl, J. Fritzenkötter, V. Gantner, R. Kaltenbach, and L. Pohl. 2020. *Community effects of cash-for-work programmes in Jordan*. Studies, 103.

[CR28] MacKinnon JG, Webb MD (2018). The wild bootstrap for few (treated) clusters. Econometrics Journal.

[CR29] Nguyen TC, Rieger M (2017). Community-driven development and social capital: Evidence from Morocco. World Development.

[CR30] Nosratabadi S, Khazami N, Abdallah M. Ben, Lackner Z, Band S, Mosavi A, Mako C (2020). Social capital contributions to food security: A comprehensive literature review. Foods.

[CR31] Pavanello S, Watson C, Onyango-Ouma W, Bukuluki P (2016). Effects of cash transfers on community interactions: Emerging evidence. Journal of Development Studies.

[CR32] Roodman D, MacKinnon JG, Nielsen MØ, Webb MD (2019). Fast and wild: Bootstrap inference in Stata using boottest. Stata Journal.

[CR33] Rothstein B (2001). Social capital in the social democratic welfare state. Politics and Society.

[CR43] Strupat, C. 2021: The preserving effect of social protection on social cohesion in times of the COVID-19 pandemic: Evidence from Kenya. DIE Discussion paper 33. 10.1057/s41287-022-00541-1 PMC9097141 35578680

[CR34] Tavits M (2009). Geographically targeted spending: Exploring the electoral strategies of incumbent governments. European Political Science Review.

[CR35] Vajja A, White H (2008). Can the World Bank build social capital? The experience of social funds in Malawi and Zambia. Journal of Development Studies.

[CR36] Valli E, Peterman A, Hidrobo M (2019). Economic transfers and social cohesion in a refugee-hosting setting. Journal of Development Studies.

[CR37] Veras Soares F, Perez Ribas R, Issamu Hirata G (2010). Impact evaluation of a rural conditional cash transfer programme on outcomes beyond health and education. Journal of Development Effectiveness.

[CR38] White, H., R. Menon, and H. Waddington. 2018. *Community-driven development: Does it build social cohesion or infrastructure? A mixed-method evidence synthesis*. http://www.3ieimpact.org/media/filer_public/2018/03/12/wp30-cdd.pdf.

[CR39] World Bank. 2020. *The World Bank in Malawi*. https://www.worldbank.org/en/country/malawi/overview.

